# The Effects of Forgiveness, Gratitude, and Self-Control on Reactive and Proactive Aggression in Bullying

**DOI:** 10.3390/ijerph17165760

**Published:** 2020-08-10

**Authors:** Fernanda Inéz García-Vázquez, Angel Alberto Valdés-Cuervo, Lizeth Guadalupe Parra-Pérez

**Affiliations:** 1 Department of Education, Technological Institute of Sonora; Obregon City 85000, Mexico; angel.valdes@itson.edu.mx; 2 School of Education, Colorado State University; Fort Collins, CO 80526, USA; lizparra@gmail.com

**Keywords:** gratitude, forgiveness, self-control, human strengths, bullying, adolescence, proactive aggression, reactive aggression

## Abstract

The social cognitive approach to moral development posits that moral self-schemas encourage character strengths and reduce adolescents’ aggression. However, limited research has examined the influence of positive personal characteristics on proactive behaviors and reactive aggression in bullying. This study examined direct and mediational relationships between forgiveness, gratitude, self-control, and both proactive and reactive aggression in bullying. The extent to which the structural relations of this model were invariant by gender and stage of adolescence were also evaluated. Participants in this study were 1000 Mexican students, 500 early adolescents (*M* age = 12.36, *SD* = 0.77 years) and 500 middle adolescents (*M* age = 16.64, *SD* = 0.89 years), between 12 and 17 years old. Structural equation and multi-group invariance analysis were performed. Results indicate that gratitude and forgiveness are positively related to self-control. Gratitude, forgiveness, and self-control are also negatively related to reactive and proactive aggression. Forgiveness and gratitude had an indirect relationship by decreasing both proactive and reactive aggression through their positive effects on self-control. Additionally, gender moderated the relationships between variables proposed in the model, whereas stage of adolescence did not. Overall findings suggest that moral self-schemas and strengths explained both types of aggression in bullying.

## 1. Introduction

Bullying is a frequent type of school violence [1,2]. Worldwide research reports that bullying incidents occur at rates from 10% to 40% of all students [3,4]. In Mexico, studies indicate that more than 20% of students between the ages of 12 and 18 are bullied [5,6]. This aggression has negative effects on students’ school engagement, well-being, and psycho-social development [7,8,9]. Bullying is defined as students’ intentional and repetitive aggression against weaker peers [10,11]. Several authors have distinguished between reactive and proactive aggression in bullying [12,13,14]. Proactive aggression is planned, unprovoked and aimed at obtaining dominance in social interactions [15,16]; while reactive aggression is an anger-driven, and often emotionally dysregulated, response to perceived offenses or frustrations [17,18].

Many studies have focused on personal risk factors related to proactive and reactive aggression [19,20,21]. However, limited research has examined positive variables that prevent proactive and reactive aggression in bullying [22,23]. Research into these variables is a relevant issue and allows better understanding of variables that decrease adolescent aggression against peers [22,24,25]. In order to close this gap, the present study adopted a social perspective on moral development [26,27] as a means to explain the relationship between moral self-schemas, reactive and proactive behaviors in bullying.

The social cognitive perspective on moral development [26,27] posits that moral self-schemas are essential to explain individual behaviors in interpersonal relationships. Moral self-schemas comprise a set of cognitive and affective perceptions about the self in moral domains [28]. These schemas encourage character strengths and prosocial behavior [29,30]. Current research also shows that moral self-schemas reduce adolescent aggression toward school peers [31,32,33]. However, most of these studies were unable to examine the different effects of moral self-schema on proactive and reactive aggression. Forgiveness and gratitude are two moral self-schemas that influence adolescent interpersonal relationships with peers. The authors posit that self-schemas are crucial to predict lower proactive and reactive aggression in bullying.

### 1.1. Forgiveness and Gratitude

Forgiveness has the potential to decrease revenge responses and provides a disposition to select situationally appropriate behavior in the context of bullying [34,35]. It involves a benevolent emotion towards the transgressor and the rehabilitation of trust and hope in relationships [31]. Forgiveness has been related to victims experiencing fewer vengeful and negative thoughts and feelings towards their aggressors [36,37,38,39], while increasing support-seeking strategies, empathy, and positive conflict resolution [34,40,41]. Some scholars have reported that increases in forgiveness are related to fewer aggressive behaviors [42,43]. Other studies show that forgiveness of bullying is associated with lower rates of peer aggression [37,44,45,46,47]. In fact, students who forgive their aggressors have been found to experience less violence [48]. However, no study known by the authors has examined how forgiveness is associated with both proactive and reactive bullying aggression.

Gratitude is the result of a positive appreciation of the benefits provided by others [49,50,51]. Some scholars suggest that gratitude has a positive relationship with wellbeing [52,53]. It also has been reported that people with greater levels of gratitude have fewer negative emotions [54,55] and more prosocial behavior [56,57]. A number of studies have shown that gratitude is associated with positive interpersonal relationships and social outcomes; however, it still remains unclear how gratitude influences negative and violent behaviors [58]. Only one other study could be found that examined the role of gratitude in bullying aggression [59]; it reported that gratitude was associated with less peer aggression among women but did not hinder bullying in men.

Overall, the literature suggests that gratitude and forgiveness are moral self-schemas that involve positive responses to harm, and can help restore relationships [60]. Some scholars have found a positive relation between these moral self-schemas and self-control in adolescents [29,61,62,63]. Despite the research that has found that self-control is a critical variable that mediates the relationships between moral self-schemas and moral behavior [64,65,66], very little is known about the relationships between forgiveness, gratitude, self-control and bullying aggression.

### 1.2. The Mediational Influence of Self-Control

Self-control is a character strength that involves the regulation or suppression of inappropriate impulses, emotions, values and actions in order to achieve a goal [67,68,69]. Self-control is necessary to allow an individual’s behavior to adjust to moral self-schemas, because self-control is relevant to overriding short-term desires, so it enables adolescents to conform to moral standards [67]. Scholars posit that self-control restrained the impulses to put the self above others, which reduces actions that are harmful to other individuals. Furthermore, self-control restrains angry behaviour derived from frustration and expressed in aggression [67,70]. 

Studies reported that high levels of self-control are related to less bullying perpetration [70,71,72,73,74]. A great deal of the literature indicates that self-control has a significant effect on proactive and reactive behaviors [18,75,76]. However, these studies are inconclusive about reactive and proactive aggression; some scholars have revealed that low self-control leads to reactive aggression [16,76,77,78], while others have reported that self-control is more strongly related to proactive than reactive aggression [75,79]. Moreover, another study reported that self-control exerts similar influences on both proactive and reactive aggression [80].

### 1.3. Moderating Role of Gender and Age

The literature evinced differences by gender and age in bullying aggression and moral self-schemas. Prior research indicates that proactive aggression was higher in males than females [18,81,82,83]. However, studies exploring the differences in reactive aggression are also inconclusive, with some research reporting that male reactive aggression was higher than female [81,82], while no gender differences were observed in others [18,83,84]. Moreover, studies suggest that females seek less revenge [34,85,86] and are more grateful than males [87,88,89,90,91]. The literature has also found differences by age. Studies report that both types of aggression decrease with age [83,92,93]. Moreover, research findings suggest that forgiveness and gratitude increase with age [92,93,94,95].

Overall findings suggest that it is important to examine whether structural relationships between forgiveness, gratitude, self-control and proactive and reactive aggression functions are similar or different across groups of adolescents. By doing so, the influence of moral self-schema and self-control on types of bullying aggression can be compared by gender and stage of adolescence.

### 1.4. The Present Study

This study examines the relationships between adolescents’ moral self-schemas, character strength, and bullying aggression. Despite the evidence suggesting that these variables prevent interpersonal aggression [37,44,59,72,74], limited studies have explored positive personal characteristics associated with bullying behavior. Moreover, although a considerable body of research has studied variables that influence bullying behavior [96,97,98], less attention has been paid to effects of these variables on both proactive and reactive aggression. Additionally, the moderated role of gender and age in the relationships between variables included in the study and both types of aggression remain unclear. Finally, research in Mexico about bullying is limited in general.

**Figure 1:**
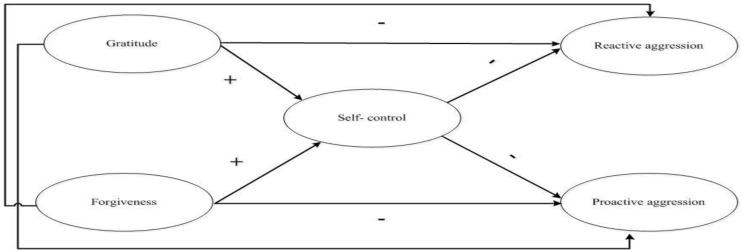
Theoretical model of the relations between gratitude, self-control, forgiveness, reactive and proactive aggression in adolescents.

**Figure 2:**
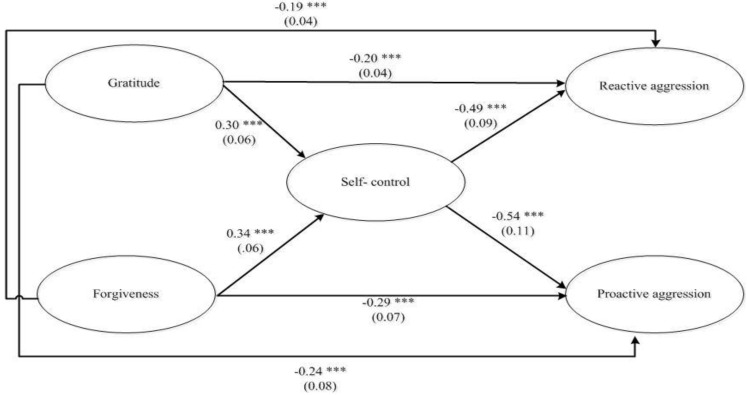
Results of the structural model of the relations between gratitude, self-control, forgiveness, reactive and proactive aggression in adolescents. Note. Standardized coefficients and standard errors are presented. *** *p* < 0.001.

In this context, this study proposed to: (1) explore direct relationships between forgiveness, gratitude, and self-control in both proactive and reactive bullying (see Figure 1); (2) analyze the mediational influence of self-control in relationships between moral self-schemas and both types of aggression; and (3) test the moderating role of gender and stage of adolescence (early vs middle) in these relations. To accomplish this, the following hypotheses were used:

**Hypothesis** **1a (H1a).** *(direct relationship with moral self-schemas): Forgiveness and gratitude were expected to have a positive relationship with self-control, and act negatively on both proactive and reactive aggression.*

**Hypothesis** **1b (H1b).** *(direct relationship with self-control): A negative relation between adolescents’ self-control and both proactive and reactive aggression was anticipated.*

**Hypothesis** **2 (H2).** *(indirect relationship): Forgiveness and gratitude have an indirect negative relation to both proactive and reactive aggression as they improve self-control.*

**Hypothesis** **3a (H3a).** *(gender moderation): Gender was anticipated to moderate the structural relationships proposed in the model.*

**Hypothesis** **3b (H3b).** *(stage of adolescence moderation): Finally, research anticipates that the stage of adolescence (early vs. middle) moderates the relationships between the study variables.*

## 2. Materials and Methods

### 2.1. Participants

The sample came from 30 public secondary and 30 high schools selected by convenience from three cities in the state of Sonora, Mexico. The study sample included 500 early adolescents (290 female and 210 male) with ages between 12 and 14 years (*M* age = 12.36, *SD* = 0.77 years), and 500 middle adolescents (260 female and 240 male), with ages between 15 and 17 years (*M* age = 16.64, *SD* = 0.89 years), who were selected by simple probabilistic sampling (*p* = 0.5, *q* = 95%). As usual in urban public schools in Mexico, this population includes middle and lower class students [99].

### 2.2. Measures

#### 2.2.1. Gratitude

*The Gratitude Questionnaire* [50] was used. The scale was validated in a Mexican adolescents’ sample [35]. According to the authors, gratitude is the positive evaluation of the profits that others provide in our life [51]. This is a one-dimension scale that comprises six items (e.g., I have many things in my life to be thankful for, α = 0.83, ω = 0.88) using a seven-point Likert scale (0 = *strongly disagree,* to 6 = *strongly agree*). The Confirmatory Factor Analysis (CFA) showed a good model fit to the data (*X²* = 10.86, *df* = 7, *p* = 0.145; standardized root mean square residual (SRMR) = 0.04; adjusted goodness of fit index (AGFI) = 0.98; Tucker-Lewis index (TLI) = 0.97; comparative fit index (CFI) = 0.98; root mean square error of approximation (RMSEA) = 0.02, CI 90 [0.01, 0.04]).

#### 2.2.2. Forgiveness

The Forgiveness *Heartland* Scale [100] was used. The scale was validated in a Mexican adolescents’ sample [35]. The authors defined forgiveness as an act that comprises the restoration of the relationship and blameless feelings toward the offender. This one-dimension scale included six items (e.g., Although others hurt me, over time I can see them as good people; α = 0.75; ω = 0.79). The seven-point Likert scale response was used (0 = *strongly disagree*, to 6 = *strongly agree*). The CFA showed a good model fit (*X² =* 33.07, *df* = 12, *p* = 0.001; SRMR = 0.07; AGFI = 0.97; TLI = 0.97; CFI = 0.99; RMSEA = 0.04, CI 90 [0.02, 0.05]).

#### 2.2.3. Self-Control

The *Short Self-Control Scale* [101] was used. The back-translations method was used for an accurate translation from English to Spanish. According to authors, self-control is the ability to change ones’ responses and interrupt undesired behavior. This unidimensional scale comprises six items (e.g., I can resist temptations; α = 0.73, ω = 0.77). The CFA supported model fit to the data (*X²* = 15.80, *df* = 9, *p* = 0.071; SRMR = 0.02; AGFI = 0.98; TLI = 0.96; CFI = 0.99; RMSEA = 0.02, CI 90 [0.01, 0.04)].

#### 2.2.4. Reactive and Proactive Aggression

The *Reactive-Proactive Aggression Questionnaire* [102] was used. The back-translation method was used for the suitability of the scale in Mexican populations. This scale included four items to measure reactive aggression (e.g., I beat others to defend myself; α = 0.80, ω = 0.82), and five items for proactive aggression (e.g., I use force to get things I want from others; α = 0.83, ω = 0.84). The CFA supported that the measurement model fitted the data (*X²* = 15.80, *df* = 25, *p* = 0.007; SRMR = 0.02; AGFI = 0.98; TLI = 0.95; CFI = 0.98; RMSEA = 0.02, CI 90 (0.01, 0.04)).

### 2.3. Procedure

The researchers gained study approval from the Research Ethical Committee of the Technological Institute of Sonora (No. 2020_0018). Later, a consent letter was sent to parents to explain the purpose of the study and to ask permission for student participation. Only 7% of the parents refused to allow their children to participate. Despite having consent letters from parents, it was explained to students that their participation was voluntary; therefore, they may withdraw themselves at any time. All of the students accepted the invitation to participate in the study. Data collection took place during regular school hours. The scales were administered by the authors with help from graduate students.

### 2.4. Data Analysis

The total percentage of missing data was 4%. In all cases, missing values were treated using the SPSS multiple imputation method. The mean, standard deviation, Pearson’s correlations and means differences by gender and state of adolescence (early vs. middle) were calculated. All CFA and structural models were calculated using AMOS. The maximum likelihood estimation (ML) with Bollen-Stine and bias-corrected confidence interval bootstraps (with 500 replicates and a 95% confidence interval) was used. The bootstrap is a robust procedure to deal with multivariate non-normality issues in structural equation modeling [103,104,105].

In order to evaluate the goodness of fit of the models, fix indices proposed by some authors were used [104,106,107,108]: (a) Chi squared and associated probability (*X^2^* with *p* < 0.001), Bollen-Stine bootstrap with *p* < 0.05, SRMR ≤ 0.08, TLI ≥ 0.95, AGFI ≥ 0.95, CFI ≥ 0.95, and RMSEA ≤ 0.05, with their confidence interval.

Finally, a multi-group analysis was performed to examined gender and stage of adolescence invariance in the relations proposed in the model. The invariance of the model was verified using indicators (Δ*X*^2^ with *p* > 0.01, ΔCFI < 0.01, and ΔRMSEA < 0.015 [104,109].

## 3. Results

### 3.1. Preliminary Analysis

Table 1 showed a significant negative correlation between forgiveness, gratitude, and self-control with both proactive and reactive aggression. Findings also suggest that forgiveness, gratitude, and self-control are positively correlated. The results show that the correlation between forgiveness, gratitude and self-control have a medium effect (*r* > 0.20). Similarly, the correlation between two moral self-schemas and proactive and reactive aggression has a medium effect (*r* > 0.20). On the other hand, the correlation between self-control and two types of bullying aggression has a larger effect (*r* > 0.30) [110]. Finally, results showed that males showed more reactive and proactive aggression, and less gratitude than females. Finally, no differences were found between early and middle adolescents in the study variables.

### 3.2. Structural Model

The structural model results are presented in Figure 2. Values of the fit indices suggest that the model had a good fit to the data (*X^2^* = 163.97, *df* = 146, *p* < 0.009; Bollen Stine bootstrap *p* = 0.04; SRMR = 0.04; AGFI = 0.97; TLI = 0.98; CFI = 0.98; RMSEA= 0.022, CI 90 (0.015, 0.028)). The model could explain 42% of the variance in proactive aggression and 37% in reactive aggression.

In Figure 2, the standardized coefficients and standard errors of the structural model are presented. The results showed that gratitude and forgiveness were positively related to self-control (β = 0.30, *p* < 0.001, CI 95% (0.22, 0.35); β = 0.34, *p* < 0.001, CI 95% (0.24, 0.41). Additionally, gratitude, forgiveness, and self-control are negatively related to reactive (β = −0.20, *p* < 0.001, CI 95% (−0.15, −0.28); β = −0.19, *p* < 0.001, CI 95% (−0.11, −0.27); β = −0.49, *p* < 0.001, CI 95% (−0.38, −0.57), respectively), and proactive aggression (*β* = −0.24, *p* < 0.001, CI 95% (−0.13, −0.37); *β* = −0.29, *p* < 0.001, CI 95% (−0.15, −0.36); *β* = −0.54, *p* < 0.001, CI 95% (−0.42, −0.60), respectively). The indirect relationship results showed that forgiveness (β = −0.13, *p* < 0.012, CI 95% (−0.04, −0.20) and gratitude (β = −0.07, *p* < 0.034, CI 95% (−0.04, −0.11) decreased proactive aggression. Furthermore, forgiveness (β = −0.11, *p* < 0.022, CI 95% (−0.05, −0.18) and gratitude (β = −0.08, *p* < 0.044, CI 95% (−0.03, −0.14) also hindered reactive aggression.

### 3.3. Invariance Analysis

Lastly, in order to examine the moderated effect of gender and stage of adolescence (early vs. middle), a multi-group analysis was performed. Testing for structural invariance includes a series of hierarchical models, beginning with the establishing of a baseline model in each group (configurational invariance), followed by testing measurement invariance and then assessing structural invariance (see Table 2).

Results showed the equivalence baseline model by gender (*X^2^* = 416.76, *df* = 278, *p* < 0.001; SRMR = 0.06; AGFI = 0.95; CFI = 0.97; TLI = 0.96; RMSEA = 0.022, IC 90 [0.018, 0.027]). However, differences in chi-squared (Δχ^2^), in the comparative fit indices (ΔCFI), and the root mean square of error (ΔRMSEA) in the structural invariance suggest that gender does moderate the relationships between variables in the model (see Table 2). The differences on structural invariance is relative to the effect of gratitude on reactive and proactive aggression. In females, gratitude is negatively related with reactive (*β =* −0.44, *p* < 0.001, CI 95% (−0.49, −0.33)) and proactive (*β =* −0.47, *p* < 0.001, CI 95% (−0.53, −0.38)) aggression, but in males these relations are not significant (*β =* −0.09, *p* = 0.34 CI 95% (−0.16, 0.07); *β =* −0.05, *p* = 0.37 (−0.11, 0.03), respectively).

However, the results do support the equivalence of baseline model to stage of adolescence (early vs. middle) in both groups (*X^2^* = 342.99, *df* = 277, *p* = 0.005; SRMR = 0.05; AGFI = 0.95; CFI = 0.98; TLI = 0.98; RMSEA = 0.015, IC [0.009, 0.020]). Also, differences in Δχ^2^, ΔCFI, and ΔRMSEA of error indicate the existence of structural invariance (see Table 2).

## 4. Discussion

Unlike most of the past studies, this research focuses on positive individual characteristics that could partially explain proactive and reactive aggression in bullying. This study explores the association of forgiveness, gratitude, and self-control with both proactive and reactive aggression in bullying. The moderate influence of gender and stage of adolescence (early vs. middle) in these relations was also examined. The findings support the hypotheses about direct and indirect relations proposed in the structural model, but only partially confirmed the moderation hypothesis.

### 4.1. Direct Relationships (Hypothesis 1a and Hypothesis 1b)

The data provided evidence that moral self-schemas are positively related to self-control. As expected, the more grateful and forgiving students were, the more likely they were to regulate their behavior and emotions. These findings are consistent with prior studies [62,63] reporting that these self-schemas may increase self-control in adolescents. Moreover, and consistent with the literature, results exhibited negative relations between gratitude and forgiveness with both types of bullying aggression [37,47,59]. In line with other scholars, we posit that forgiveness and gratitude hinder negative affect (for example, revenge and anger) [39,56,70,71], while encouraging positive affect (for example, compassion and empathy) [111,112,113,114]. The medium effect size indicates that this is of explanatory and practical use in the short run [110].

Furthermore, the results suggest that self-control is a relevant strength that decreases proactive and reactive aggression. These findings are consistent with the findings of previous studies that self-control reduces proactive and reactive aggression [75,79]. In line with other scholars, we believe that moral self-schemas improve adolescent self-control because, with these, the adolescent does not turn a perceived aggression into possible action [115,116]. The larger effect-size suggest that this is powerful in both the short and long run [110].

### 4.2. Mediational Influences (Hypothesis 2)

The results showed that adolescents’ self-control partially mediated the relationship between gratitude and forgiveness and both types of aggression. To be specific, these moral self-schemas appear to enhance self-control, and thus decrease reactive and proactive aggression. Our results are consistent with studies that show self-control is an important variable that mediates the relationships between morality and moral behavior [64,65,66]. In line with other scholars, we believe that both moral self-schemas and self-control are implicated in moral behavior because while moral self-schemas guide the perception of moral behavioral options, self-control inhibits perceived non-moral action alternatives [117].

### 4.3. Gender and Age Moderation (Hypothesis 3a y 3b)

Findings revealed that gender did moderate the relationships proposed in the structural model. Similar to previous studies, we found that gratitude is related to lowering both types of aggression in females, but not in males [59]. We believe that these findings should be explored further to study differences in perception of gratitude of both genders. Scholars suggest that cultural context may influence the idea that gratitude is experienced as a weakness by males, and consequently men avoid expressing gratitude in interpersonal relationships [118,119,120].

Finally, the study showed that adolescence stage did not have a moderating effect on the relationships proposed in the model. This means that the influence of forgiveness, gratitude and self-control on proactive and reactive aggression was similar in early and middle adolescents. These findings are contradictory to the current literature [83,86,93,94,95]. Although additional studies are necessary to elucidate the moderate effects of age on the proposed relationships, we believe that these findings may be explained by the fact that Mexican culture emphasizes strong protection and dependence on parents throughout adolescence.

### 4.4. Theoretical and Practical Implication

From a theoretical perspective, these results showed that a social cognitive perspective on moral development [26,27] is a suitable framework to study how morality is associated to aggression in bullying. In particular, the studies evinced that moral self-schemas [27] are valuable constructs that encourage character strengths and reduce proactive and reactive aggression in adolescents. Moreover, these findings suggest that self-control is a critical variable to explain associations between morality and moral behavior in adolescents.

From a practical perspective, the findings suggest that promoting forgiveness and gratitude could be a useful path when designing school interventions to reduce bullying aggression. According to these results, we consider it relevant to explore positive characteristics that may help decrease bullying, instead of focusing only on risk factors. Hence, it is necessary to offer adolescents an adequate environment that provides the opportunity to develop and display these positive characteristics through relevant tasks and settings.

### 4.5. Limitations

The first limitation of the study was that a cross-sectional design does not allow the probing of causal associations among variables. Therefore, other experimental or longitudinal designs are recommended to explore more deeply the relationships between variables. Second, measurements were made through self-reports. Although, the scales have adequate psychometric properties, studies using multiple measures are suggested. Third, despite the relatively large sample, in order to generalize the results it is necessary to pursue more diverse samples.

## 5. Conclusions

In conclusion, the results of this research contribute to understanding the different effects of moral-self schemas on proactive and reactive aggression and supports the idea that gender moderates the relations proposed in the model. Findings demonstrated that forgiveness and gratitude directly and indirectly decrease proactive and reactive aggression in bullying. However, the medium effect size of these relationships suggests a practical value, in particular, in explaining the decrease in student’s aggression in the short run. Furthermore, our results indicate that self-control was associated to a lower level of both proactive and reactive aggression in adolescents. The large effect size of these relationships indicates that self-control has a powerful short term and long term effect in hindering both proactive and aggressive aggression in bullying.

In addition, prevention approaches and programs should consider gender differences in the appreciation of benefits provided by others, due to the stronger effect of gratitude in both reactive and proactive aggression in females. Finally, prevention efforts could be more effective if they focused on developing moral and positive characteristics that help conflict resolution and avoid aggressive behavior in adolescents.

## Figures and Tables

**Table 1:** Means, standard deviations, correlations, and mean comparisons by gender and stage of adolescence.

Variable	*M*	*SD*	1	2	3	4	5
1. Reactive aggression	1.06	0.75	-				
2. Proactive aggression	0.53	0.37	0.55 **	-			
3. Gratitude	4.74	1.39	−0.27 ***	−0.19 ***	-		
4. Self-control	2.69	0.78	−0.41 ***	−0.37 ***	0.27 ***	-	
5. Forgiveness	2.75	1.18	−0.24 ***	−0.28 ***	0.20 ***	0.28 ***	-
*M/SD* Male			1.23/0.76	0.55/0.43	4.50/1.52	2.64/0.78	2.70/1.27
Female			0.90/0.70	0.47/0.38	4.94/1.23	2.72/0.77	2.80/0.76
Student’s *t*			7.16 ***	4.63 ***	−5.07 ***	−1.51	−1.08
Cohen‘s *d*			0.45	0.28	0.31	0.10	0.09
*M/SD* Early adolescent			1.07/0.73	0.53/0.30	4.80/1.34	2.70/0.78	2.76/1.37
Middle adolescent			1.03/0.76	0.55/0.35	4.68/1.43	2.68/0.78	2.78/1.40
Student’s *t*			0.82	−0.27	1.36	0.33	0.04
Cohen’s *d*			0.05	0.06	0.08	0.02	0.01

** *p* < 0.01. *** *p* < 0.001.

**Table 2:** Results of the invariance analysis by gender and stage of adolescence (early vs. middle).

Invariance Models					
Gender	Δχ^2^	Δ*df*	*p*	ΔCFI	ΔRMSEA
Measurement weight	30.34	14	0.070	0.003	0.001
Structural weight	51.58	20	<0.000	0.016	0.02
Stage of adolescence					
Measurement weight	12.97	14	0.538	0.001	0.000
Structural weight	16.41	20	0.690	0.000	0.001
